# High-quality genome assembly and annotation of *Clonostachys chloroleuca* strain Cc878 using Oxford Nanopore long-read sequencing

**DOI:** 10.1128/mra.00357-24

**Published:** 2024-06-20

**Authors:** Xin Zhang, Xinyuan Long, Xiaoxing Xing, Jia Tai, Guanghui Wang, Ming Xu, Huiquan Liu

**Affiliations:** 1 State Key Laboratory for Crop Stress Resistance and High-Efficiency Production, College of Plant Protection, Northwest A&F University, Yangling, Shaanxi, China; University of Guelph, Guelph, Canada

**Keywords:** *Clonostachys*, genome assembly, gene annotation

## Abstract

As a noteworthy biocontrol fungus, *Clonostachys chloroleuca* currently lacks a high-quality reference genome. Here, we present the first high-quality genome assembly of *C. chloroleuca* strain Cc878 achieved through Oxford Nanopore Long-Read sequencing. The nuclear genome of Cc878 was assembled into four contigs, totaling 59.38 Mb.

## ANNOUNCEMENT

*Clonostachys* spp. are biocontrol fungi with saprotrophic, mycotrophic, and plant endophytic lifestyles (1). These fungi can directly antagonize plant pathogens, promote plant growth, and induce resistance to protect plants from disease (2–4). While a high-quality genome assembly is available for the well-known species *C. rosea*, it is rare for other species in *Clonostachys*. Currently, the genomic assemblies for *C. chloroleuca* are limited to strains 67-1 and CBS 570.77, which were generated using Illumina technology (5, 6). These genome assemblies contain hundreds of contigs, diminishing their value as high-quality reference genomes for studying the molecular level of biocontrol mechanisms and conducting comparative genomics.

The *C. chloroleuca* strain Cc878 was originally isolated from soybean rhizosphere soil in Harbin City, Heilongjiang Province, China. The confirmation of Cc878 as *C. chloroleuca* was established through phylogenetic analysis of the ATP citrate lyase gene (GenBank: KX184855.1).

**Fig 1:**
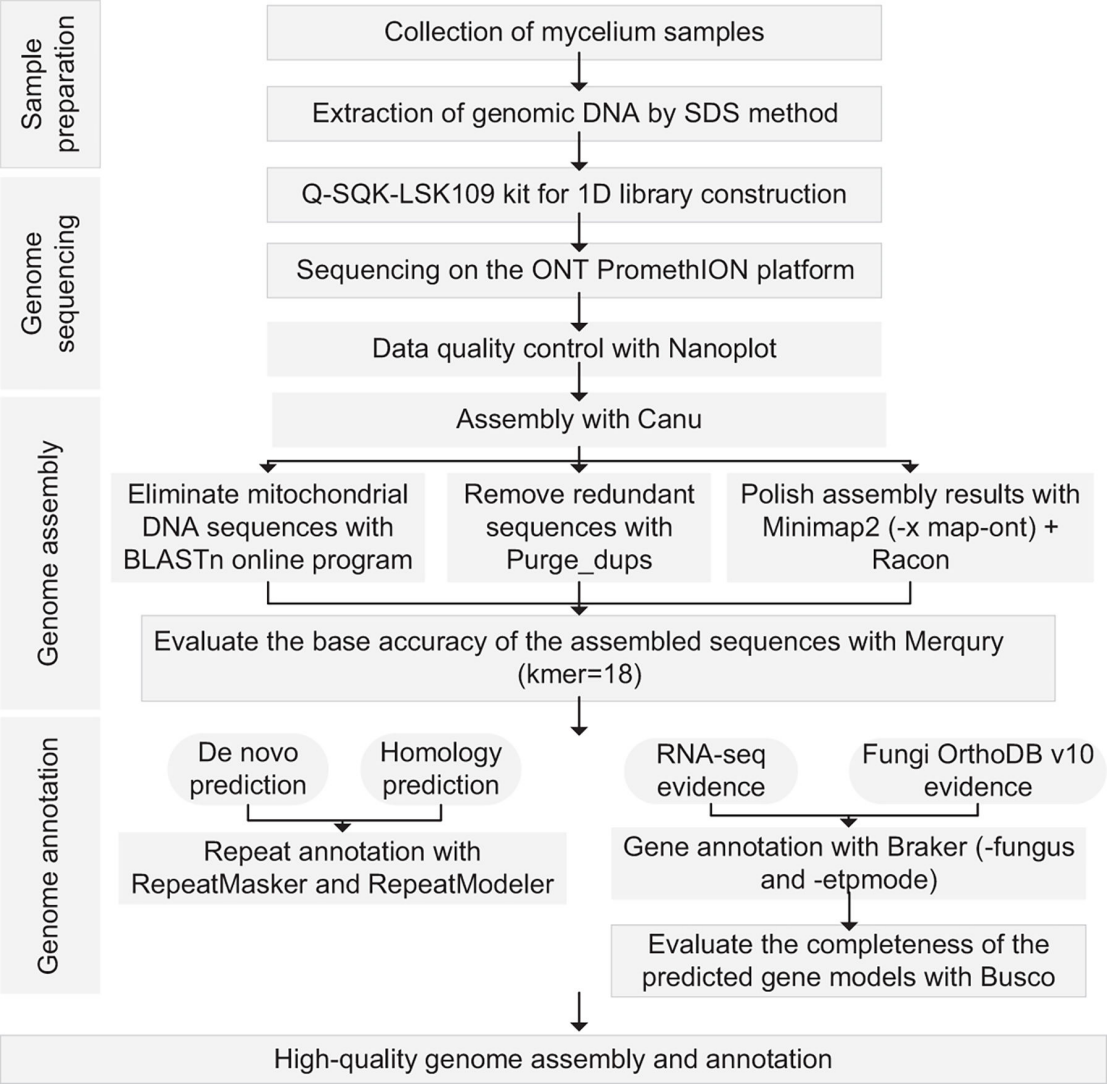
A schematic diagram that outlines the workflow for sample preparation, genome sequencing, assembly, annotation, and subsequent evaluation of the Cc878 genome.

To provide a high-quality reference genome, we conducted *de novo* sequencing and assembly of the Cc878 genome using Oxford Nanopore Technology (Fig. 1). Cc878 was cultured on potato dextrose agar (PDA) medium at 25°C for 10 days. Spores were harvested by washing and filtering with sterile water. The spores were then used to inoculate yeast extract peptone dextrose (YEPD) medium to grow the mycelium. Genomic DNA was extracted using the SDS method (7), and its quality and quantity were assessed by agarose gel electrophoresis and Qubit 2.0 Fluorometer (Thermo Scientific), respectively.

Libraries were constructed with an insert size of ≥20 kb using a 1D ligation sequencing kit (catalog no. Q-SQK-LSK109) and sequenced on the PromethION platform. Base calling was performed using Guppy v6.2.7 (8), with the parameter “-c dna_r9.4.1_450bps_hac_prom.cfg.” A total of 1,352,886 filtered reads (10.31 Gb; 161× coverage) were obtained, with a mean length of 7,618 bp and an N_50_ length of 10,471 bp.

The read correction, trimming, and genome assembly of Cc878 were conducted using Canu v2.2 (9) with default parameters. Mitochondrial DNA sequences were eliminated using BLASTn. Purge_dups v1.2.6 (10) was employed to remove redundant contigs in the primary assembly. To enhance assembly accuracy, long reads were initially aligned back to the assembly using Minimap2 v2.24 (11) with the parameter “-x map-ont” and the error correction was then performed using Racon v1.4.3 (12). Subsequently, the contigs were scaffolded using RagTag v2.1.0, and assembly quality was assessed using QUAST v5.0.2 (13) with default parameters.

The final genome assembly of Cc878 was 59.38 Mb, consisting of four contigs with an N_50_ of 37.47 Mb. The assembly had a GC content of 48.76% and a repeat content of 7.43%. According to Merqury V1.3 (14) assessment, the assembly had a consensus quality (QV) score of 45.75, corresponding to 99.99% accuracy at Q40, and a k-mer completeness score of 98.49%.

The BRAKER2 gene prediction pipeline was employed to predict genes using transcriptome evidence (RNA-seq data from *in vitro* vegetative growth and *in planta* saprophytic/endophytic growth) and homologous protein evidence (fungi OrthoDB v10 database) (15). Overall, we identified 21,504 protein-coding genes (>50 amino acids, Busco completeness score 98.94%) in the Cc878 genome.

## Data Availability

The whole-genome sequence has been deposited at DDBJ/ENA/GenBank under the accession number JBANAY000000000. The raw reads have been deposited in the SRA under the accession number SRR27765574.
